# From expression cloning to gene modeling: The development of *Xenopus* gene sequence resources

**DOI:** 10.1002/dvg.22008

**Published:** 2012-02-16

**Authors:** Michael J Gilchrist

**Affiliations:** Division of Systems Biology, MRC National Institute for Medical Research, The Ridgeway, Mill HillLondon NW7 1AA, United Kingdom

**Keywords:** full-length clones, cDNA, gene modeling, mRNA sequence, EST assembly, genome assembly

## Abstract

The *Xenopus* community has made concerted efforts over the last 10–12 years systematically to improve the available sequence information for this amphibian model organism ideally suited to the study of early development in vertebrates. Here I review progress in the collection of both sequence data and physical clone reagents for protein coding genes. I conclude that we have cDNA sequences for around 50% and full-length clones for about 35% of the genes in *Xenopus tropicalis*, and similar numbers but a smaller proportion for *Xenopus laevis*. In addition, I demonstrate that the gaps in the current genome assembly create problems for the computational elucidation of gene sequences, and suggest some ways to ameliorate the effects of this. genesis 50:143–154, 2012. © 2012 Wiley Periodicals, Inc.

## INTRODUCTION

*Xenopus* is an excellent model system for the study of early development in vertebrates, with its accessible and easily manipulated embryos. Unraveling the molecular biology of development depends on having a good knowledge of the molecules involved; and probably the most significant group of these are the protein coding genes. Vertebrate protein coding genes are generally multi-exonic, having a relatively short 5′ UTR, a rather longer 3′ UTR, and the ability to express different combinations of exons as alternative transcripts. The degree to which the sequences of these genes are accurately defined in our databases will largely determine the ease with which we can progress experimentally. Although the sequence of a gene may be thought of in a number of different ways, a great deal can be accomplished knowing only the mRNA sequence(s) of a gene, and here I concentrate largely on this.

One of the key advantages of the *Xenopus* system is the ability to generate protein directly in the early stage embryo by simple injection of mRNA, and then observe the result of the consequent *over-expression* of the gene (Smith and Harland, 1991; Voigt *et al*., 2005). Here we see two divergent but highly complementary needs for the ‘gene sequence’: (a) *knowing* the sequence of the mRNA and having it available and annotated in databases, and (b) *having* the mRNA sequence suitably captured in a physical clone-based reagent that can be used directly in experiments.

Other advantages of the *Xenopus* system have been well reviewed (Amaya, 2005; Carruthers and Stemple, 2006; Harland and Grainger, 2011), but in this context it is worth highlighting those which depend on the completeness and quality of the expressed sequence resources in some way. It is noticeable that the way these methods have developed both drives and mirrors the improvement in gene sequence resources that are being reporting on here. In the type of over-expression, or gain-of-function, screens described above, the early approaches screened through large numbers of unsorted clones from cDNA libraries (Smith and Harland, 1991, 1992), whereas later ones (Chen *et al*., 2005; Voigt *et al*., 2005) were able to benefit from computational analysis of large EST sequence data sets (Gilchrist *et al*., 2004), to reduce redundancy and provide *a priori* identification of the genes involved.

Loss-of-function effects can also be studied efficiently in *Xenopus*, using antisense morpholino oligonucleotides to knock down gene function (Chang *et al*., 2006; Sander *et al*., 2007). For this technique precise knowledge of the gene sequence is required: the true initiator methionine for translation blocking, and the position and sequence of exon boundaries for splice interference. The method is well suited to both small (Kenwrick *et al*., 2004) and medium sized (Rana *et al*., 2006) screens, and clearly provides a cost- and time-effective approach to loss-of-function screens in a vertebrate system, as well as a tool for detailed dissection of individual gene function. It can also be used to perform multiple simultaneous knock-downs in the same embryo, with little increase in experimental complexity (Khokha *et al*., 2005). This compares favorably to the process of creating and combining true gene knock-outs in other vertebrates.

Expression arrays can yield valuable information about coordinated changes of gene expression, and, in a similar way to the use of over-expression screens described above, these have become more sophisticated and comprehensive over time. Early efforts used randomly selected cDNAs from a library of interest to populate an array (Altmann *et al*., 2001), whereas later arrays, using data from the large-scale sequencing projects, could assay gene expression over much of the developmental repertoire (Chalmers *et al*., 2005; Langerveld *et al*., 2009).

More recent developments of high throughput, antibody-based ChIP assays have allowed detailed investigation of gene regulatory mechanisms. These have a high requirement for complete gene sequence information, to define the spatial relationship between ChIP peaks and gene loci on the genome sequence. ChIP has classically been done with microarrays, but there is now a strengthening shift to the use of high throughput sequencing (HTS) technologies. Typical areas of application are: single protein binding assays to locate direct targets of a transcription factor being investigated (Blythe *et al*., 2009); binding assays of a member of the basal transcription mechanism (typically RNA-pol-II) to investigate large scale gene transcriptional activation (Akkers *et al*., 2009); and DNA modification assays to investigate chromatin state and hence gene activation and/or suppression through epigenetic markers (Akkers *et al*., 2009).

A subtler requirement for our definition of gene sequences concerns the untranslated regions of mRNAs, which contain many of the signals for posttranscriptional regulation (Mignone *et al*., 2002). For example, the precise upstream boundary of the 5′ UTR defines the edge of the proximal promoter region within which are found some of the most important signals for transcriptional activation, and the 3′ UTR is the main focus for target prediction of micro-RNA binding sites, increasingly seen as important in posttranscriptional gene regulation (Agrawal *et al*., 2009; Qiu *et al*., 2009).

It is clear that the better we define the gene sequences, the better and the more comprehensively we will be able to do good research in this highly attractive model system. The purpose of this review is to find out how close we have come to defining the full complement of *Xenopus* gene sequences, and for what proportion of these we have a physical reagent available.

## VERY DIFFERENT SOURCES OF mRNA SEQUENCE INFORMATION

There are three rather different sources of useful gene sequence data: (i) full-insert sequencing of single mRNA molecules from a cloned cDNA, (ii) EST contig assembly from large scale end-sequencing of cDNA libraries, and (iii) gene modeling on assembled genome sequence. I will look briefly at the characteristics of each method, as they have different impacts on the nature and quality of the data we have access to.

- Full-insert cDNA sequencing: Full-insert sequencing is expensive, but accurate, and gives full coverage of the cloned molecule. Cloned gene sequences may arise from within individual laboratory research programs, or be selected bioinformatically from the analysis of large-scale EST sequencing projects (Gilchrist *et al*., 2004; Morin *et al*., 2006). Individual cDNA sequences are computationally assembled from multiple single-pass sequence reads, and can generally be relied on. Such sequences are suitable for direct submission to public sequence repositories such as GenBank.
- EST contig assembly: Although, on their own, EST sequences are not generally of high enough quality to use as a reliable source of gene sequence data, in combination they can be very powerful. Large collections of EST sequences may be *clustered* (grouped according to gene) and then assembled into gene or transcript sequence *contigs* (assemblies of continuously overlapping sequences) (Gilchrist *et al*., 2004; Nagaraj *et al*., 2007; Scheibye-Alsing *et al*., 2009). A consensus sequence can be derived from such an EST contig assembly, and this should correspond to the mRNA sequence of the gene. This process is made more complicated in the presence of alternative transcripts, and various types of mis-assembly are also possible.
- Gene modeling: Given a long enough stretch of genomic DNA sequence it is possible to infer the presence and position of coding exons within the sequence. It is harder to identify the limits of UTR regions outside of the coding exons. Gene modeling is amenable to computational implementation (reviewed in Picardi and Pesole, 2010), and in the best cases it can be very accurate. It is, however, dependent on the quality and completeness of the genome assembly being used, and can also be misled by pseudo genes, local genome duplications, short tandem repeat sequences, and sections of transposable element sequence. The result of gene modeling is a collection of computationally derived transcript sequences, often limited to the coding region of the gene. These sequences are routinely submitted to the public sequence repositories, although sequence data derived in this manner is usually indicated explicitly; for example the NCBI RefSeq (Pruitt *et al*., 2007) data, where accession numbers with XM_ and XP_ prefixes show this.

**Fig 1:**
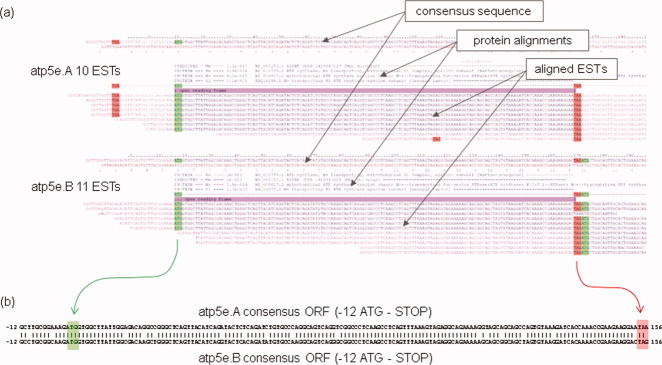
Using EST assemblies to accurately define transcript sequences. (a) EST contigs for a pair of *Xenopus laevis* homeolog genes (H+transporting F1 ATP synthase, epsilon subunit, atp5e), illustrating sensitivity of clustering and accuracy of assembly. The EST assembly for each homeolog is shown, where the consensus sequence is constructed from the aligned EST sequences. Protein alignments are used to determine the frame and position of the ORF. (b) BLASTn alignment of the open reading frames (plus 12 bases upstream) of these two genes illustrates how important knowing both these sequence is to the process of morpholino design. The Gurdon IDs for the assemblies for the A and B homeologs are Xl2.1-LANE.XL433b16.5 and Xl2.1-Ls19H.BX846122.5, respectively.

Of these three sources of sequence data, that from EST assemblies does not, by convention, find its way directly into the public databases. EST assemblies remain an important source of information (e.g., for clone selection), and current approaches can deliver data with useful sensitivity and accuracy. Consider the EST assemblies for the two *Xenopus laevis* homeologs (also known as allo- or pseudoalleles) of the gene **atp5e** (H+ transporting F1 ATP synthase, epsilon subunit) from the EST database at http://genomics.nimr.mrc.ac.uk/apps/ESTs (see Fig. 1). The 21 ESTs appear to have been correctly assembled into two clusters with ∼90% identity between the two consensus sequences. Further searching at NCBI suggests that we only have the full-length cDNA for one of these genes. In this case, the existence of the EST assemblies allows us to identify that we would need to design two morpholinos to knock down the gene function of these similarly expressed genes, whose functional overlap is not fully understood.

## INITIAL IMPETUS CAME FROM GENE CLONING IN *Xenopus laevis*

**Fig 2:**
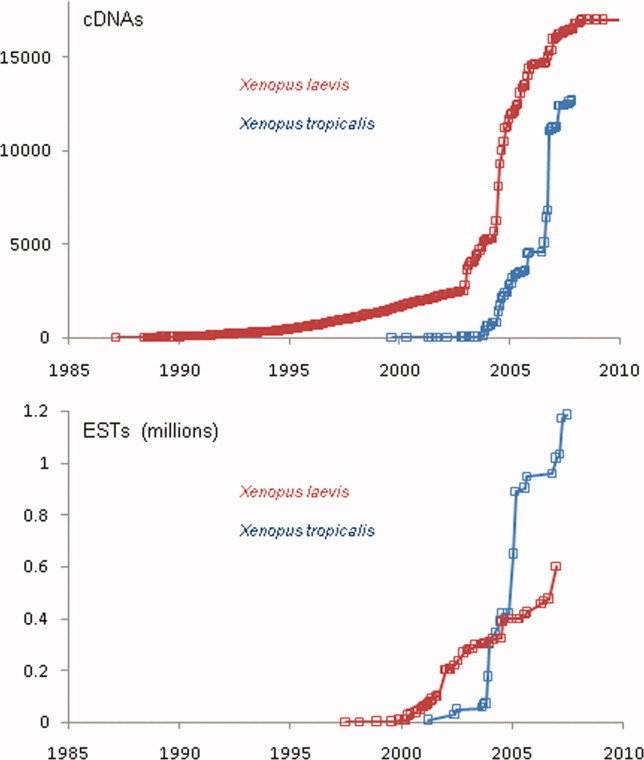
Accumulation of *Xenopus* cDNA and EST sequences over time. Computational analysis of sequence submission dates reveals the rates of arrival of different types of gene sequence for the two frogs. Upper panel: cDNA numbers analyzed by individual submission date pooled by month. Lower panel: EST numbers grouped by UniGene library entry, using earliest submission date noted for each library, and pooled by month.

The allotetraploid (from the hybridization of two similar but nonidentical genomes) *Xenopus laevis* has been a model system for much longer than its smaller, diploid cousin *Xenopus tropicalis*, and the earliest gene sequences were elucidated in the larger species by the painstaking process of expression- or library-based cloning for genes of interest. This can be seen very clearly in the steady, but clearly not exponential, rate of growth in the numbers of cDNA sequences for *X. laevis* (see Fig. 2) over the period 1987–2002 (Dale *et al*., 1992; Fritz and De Robertis, 1988; Smith and Harland, 1992 for example).

During this first period the *Xenopus* community published around 2,500 full-length mRNA sequences, which would also have been made available in physical clone form. It is not clear how many genes this represents, as there is likely to have been a degree of duplication in the research efforts of different groups. This clearly shows that a substantial number of developmentally important genes had been sequenced in this ‘pregenomic’ period.

Towards the end of this period the game changed considerably: first with the introduction of *X. tropicalis* into the equation, and then with the advent of large-scale EST projects and their consequent full-length sequencing programs, although these were by no means independent events. The introduction of *X. tropicalis* was predicated on two assumptions: one, that developmental biology in general, and the *Xenopus* community in particular, would benefit from a genetically tractable amphibian model organism; and two, that it would be relatively tough to assemble the genome sequence of *X. laevis*, with its notionally duplicated genome. In retrospect the difficulties of assembling the *X. laevis* genome may have been more imagined than real, but it would have been a larger and probably more expensive project.

By October 2004 we had a publicly released version of the *X. tropicalis* genome assembly (v3.0), shortly followed by v4.1 in August 2005 (Hellsten *et al*., 2010), which has remained the public version to the date of writing. The community has gained a suitable ‘genetic’ model organism, with an assembled genome sequence and all the avenues of research that opens up. The ‘other’ *Xenopus* continues to be much valued for its robustness under experimental conditions, and the ease with which it can be kept and handled. Different frogs for different courses, to faintly echo the introduction to a useful review of the relative merits of these two models (Harland and Grainger, 2011). The community would very much like the complete genome sequence of both frogs, and the timely advent of HTS technology suggests that we may make good progress in this direction.

## DISCOVERY RATE TRANSFORMED BY LARGE-SCALE EST PROJECTS

Prior to the arrival of the genome assembly efforts were underway in the community to generate large-scale expressed sequence data. This had the explicit aim of improving the *physical* clone resource, as well as the general aim of defining the repertoire of mRNA sequences in *Xenopus*. These began to deliver sequence data at about the same time as the early genome assemblies were made.

These large-scale EST projects represented a major change in the way the community collected full-length clones and cDNA sequences. The basic rationale was to generate hundreds of thousands of EST sequences from a small number of carefully chosen cDNA libraries, which would capture mRNAs from the widest possible range of different genes. The clustering and assembly of these EST sequences then allowed the bioinformatic prediction of large numbers of clones which should contain full-length coding sequence. This made possible the systematic selection of a nonredundant set of clones for full-length sequencing and inclusion in physical full-length clone collections.

This switch from individual laboratory to community based generation of clones and sequences can be seen very clearly in the data. There are steep rises in the numbers of ESTs in 2002 (*Xenopus laevis*) and 2004 (*Xenopus tropicalis*), followed a year or two later by large increases in the numbers of cDNAs (see Fig. 2). Subsequent steep rises reflect the iterative nature of these projects, as initial batches of finished cDNAs and new batches of ESTs allowed further rounds of full-length prediction and sequencing.

There were two main efforts using this large-scale prediction and sequencing approach: one in the UK funded by the Wellcome Trust, involving scientists from (what is now) the Gurdon Institute in Cambridge and the nearby Sanger Institute (Gilchrist *et al*., 2004); and one in the US funded by the NIH, involving scientists from the IMAGE Consortium Xenopus Gene Collection, the Joint Genome Institute, and many individual labs (Klein *et al*., 2002). Although, these two projects were initially uncoordinated, they were brought together under the auspices of the NIH supported *Xenopus* resources group (Klein *et al*., 2002) in order to reduce duplication of effort, improve the utility of the physical clone set by suitable choice of cloning vector, and perform bioinformatic analysis that would guide further choice of cDNA library input material to maximize diversity of captured genes.

These data can be seen from a different perspective if we look at the names associated with the sequence submission process for entry of the cDNAs into the public databases. Computational extraction of the last author from the GenBank records for these sequences allows cDNA submissions to be counted by principal investigator. If last authors are ranked by decreasing numbers of submissions we see the large-scale projects at the top followed by individual labs (see Table 1), and we also see a clear difference between the two species in terms of the numbers of individual laboratory submissions.

**Table 1:** Analysis of cDNA Numbers by Last Author (for the First Listed Publication) in GenBank Entries

*Xenopus laevis cDNAs*		*Xenopus tropicalis cDNAs*	
13,273	Richardson, P.	6237	Richardson, P.
218	Niehrs, C.	6041	Zorn, A. M.
48	Pieler, T.	362	Marra, M. A.
43	Litman, G. W.	6	Hedrick, J. L.
42	Knochel, W.	3	Isaacs, H. V.
38	Krieg, P. A.	3	Asashima, M.
38	Kirschner, M. W.	3	Kelley, D. B.
37	De Robertis, E. M.	2	King, M. L.
36	Asashima, M.	2	Amaya, E.
35	Dawid, I. B.	1	Adolph, K. W.
34	Goris, J.	1	Kampe, O.
34	Yoshizato, K.	1	Broders, F.
33	Strausberg, R.	1	Destree, O.
30	Du Pasquier, L.	1	Destree, O. H.
29	Haire, R. H.	1	Zhao, S.
28	Taira, M.	1	Meyerhof, W.
25	Brandli, A. W.	1	Nicholls, R. D.
24	Brown, D. D.	1	Papalopulu, N.
24	Steiner, L. A.		
24	Ueno, N.		
23	Moon, R. T.		
23	Flajnik, M. F.		
22	Harland, R. M.		
22	Pinder, A.		
22	Taranin, A. V.		

These data show very clearly the different roles played by both *Xenopus* species, and by large-scale screens, in building up our collections of full-length cDNAs. Data shown for both species, first column is the number of cDNAs attributed to each last author, and second column is the author's name. Rows are ordered by decreasing numbers of submissions, showing only the first 25 most prolific submitters. Data computationally mined from NCBI GenBank data format for all (non-Refseq) cDNAs held in our EST/cDNA database as of August 2011.

## HOW MANY GENES WERE FOUND?

The large-scale EST/cDNA projects were wound down in 2007, and their impact on our collection of reagents and sequences can now be assessed. The IMAGE/XGC clone set consists of 104 × 96-well plates for *Xenopus tropicalis* and 54 × 96-well plates for *Xenopus laevis*, and the Wellcome/Sanger set 96 × 96-well plates for *X. tropicalis*. This adds up to 14,400 and 9984 clones for the smaller and larger frogs, respectively. This does not, however, translate into the same number of unique genes. This is partly because of redundancy between and within the sets, and partly because some of the clones did not contain correct full-length sequences. In addition, the Wellcome/Sanger set was extended with repicks for barren wells discovered in the main distribution, so some genes were knowingly duplicated.

The IMAGE/XGC clones were sequenced and analyzed before final rearraying and distribution, whereas the Wellcome/Sanger clones were distributed before being resequenced, and this shows up strongly in the subsequent cDNA QC analysis (see Table 2). Around 30% of the Wellcome/Sanger clones ultimately failed to give a full-insert cDNA sequence. Of those which were successfully sequenced, just over 70% were classified as full-length. The equivalent figure for 24 of the 54 *X. tropicalis* plates analyzed from the IMAGE/XGC set was over 90% full-length. Given the consistency of the IMAGE methods, we can reasonably assume that this figure would be the same for the whole set.

**Table 2:** Analysis of Full-Insert cDNA Sequences from Full-Length Clones Sets

Wellcome/Sanger	IMAGE/XGC	Code	Description
2772	4	?	(lacks full-length sequencing result)
64	8	CHI	Chimeric Clone
4585	1795	FL	Full-Length
256	31	FLq	Possibly Full-Length
147	35	FLsh	Odd Short Form
715	0	FLt3	Truncated 3-prime
335	3	FLt5	Truncated 5-prime
228	3	FLx	Probably Non-Coding
96	37	FS	Frame Shifted
18	4	IMM	Immature/mis-spliced mRNA
9216	1920^*^		^*^data incomplete

Analysis of results of full-insert sequencing of full-length clones collections. cDNA sequences are analyzed by alignment against assembled EST contigs and tested for extent as compared to the contig ORF, and for frame-shift, sequence divergence, and other errors, against the contig consensus sequence.

From these data we can get at the *numbers of genes* for which we have a full-length clone. Taking the accession numbers of all the sequences generated from clones in the three sets, these were compared to data downloaded from the NCBI Gene database (http://www.ncbi.nlm.nih.gov/gene), which records accession numbers and Gene IDs. This gives us a count of the number of genes in each clone set (or species) for which we have one *or more* clones. Factoring in the explicit or estimated full-length rates presented above, it tells us that we are likely to have full-length clones for ∼8,380 *X. laevis* genes and ∼6,620 *X. tropicalis* genes in these physical reagent sets (see Table 3). For reasons which are not clear from the data, there are 373 cDNAs annotated as full-length from the Wellcome/Sanger set that do not have entries in the NCBI Gene database, but even adding these to our total there is clearly plenty of scope to extend these resources. These numbers suggest that we have full-length clones for around 35% of the *Xenopus tropicalis* protein coding genes, and a larger number, but probably a smaller proportion, for *Xenopus laevis*.

**Table 3:** Analysis of Gene Counts from Full-Length Clones Sets

Species	Source	Plates	Wells	Distinct clones	Distinct genes	Estimated genes with FL cDNA
*Xenopus tropicalis*	IMAGE/XGC	54	5184	5165	4973	4476^a^
*Xenopus tropicalis*	Wellcome/Sanger	96	9216	5293	4911	3904^b^
*Xenopus tropicalis*	(combined sources)	150	14,400	10,458	7681	6619^c^
*Xenopus laevis*	IMAGE/XGC	104	9984	9671	9256	8381^a^

Comparison of accession numbers for the different sets of full-length clones with entries in the NCBI Gene database indicates that gene coverage is still some way below 50% for both frog species.

a Estimated 90% FL from sample analysis of 24 plates.

b With cDNAs explicitly analyzed as FL.

c Combination of explicit and estimated analyses.

Turning to numbers of sequences, we would like to know for what proportion of genes we have the full-insert cDNA sequence; and for this we need to know how many genes we expect there to be. For *X. tropicalis* we estimate there to be 20,000–21,000 protein coding genes using “homology based gene prediction methods and deep [expressed sequence] resources” (Hellsten *et al*., 2010). This is pretty much in line with other diploid vertebrates. It is more difficult to estimate gene count in *X. laevis* because of the presumed tetraploid duplication, but we would reasonably expect at least half as many active genes again.

Although, there are over 26,000 cDNAs for *X. tropicalis* in the databases, this does not at all suggest complete coverage, as there is much duplication. Some of this duplication is incidental, as the same gene is sequenced in different projects, and some is structural. For example, the wholesale conversion of sequences from general GenBank submissions to RefSeq entries has created many simple duplicates. I obtained a complete list of cDNA accessions from the NCBI UniGene (Sayers *et al*., 2011) database (build 47, downloaded January 2009), and combined this with the NCBI Gene database to estimate gene coverage. There are 22,472 entries in the Gene database for genes with associated proteins, but only 10,549 of these have accession numbers for cDNAs attached to them. Depending on which estimate of gene count you take, this suggests that we have cDNAs for between 46 and 52% of protein coding genes in *X. tropicalis*.

It is therefore clear that we do not have full-length, cDNA-derived sequences for a large proportion of genes. Can we make up the balance by mining the genome sequence through gene modeling?

## THE IMPACT OF THE GENOME

Although, there were a number of earlier assemblies resulting from the *Xenopus tropicalis* genome project, the most widely used assembly has been v4.1, which was released in 2005 (Hellsten *et al*., 2010). Unlike the highly finished genomes of human, mouse, fly and worm, this assembly (like many of its contemporaries) is relatively incomplete (∼95%), having large numbers of gaps in the assembled sequence. The genome sequence is available as 19,759 scaffolds, constructed from ∼175,000 sections of continuous sequence, linked together where possible by large-insert clone-end data. Gaps within the scaffolds, i.e. between sections of sequence, are represented by inserted N's, and the total length of elucidated sequence is ∼1.5 billion base pairs.

Simple arithmetic indicates that the average distance between gaps within scaffolds is about 8.6 kb. When we consider that multi-exon gene loci are typically tens of kb long, we can see that gaps will routinely be found in gene loci, and thus have the potential to disrupt the gene modeling process. So while the genome is an extremely valuable resource for large-scale research, its current structure places some limits on the work it makes possible.

There are two rather different ways in which the gaps affects our ability to find and define gene loci: (i) the gene locus is sufficiently disturbed, or even absent, so that gene modeling fails, and (ii) gene modeling takes place but the gaps in the genome assembly sequence lead the programs into making poor choices, and partially incorrect sequences are generated. The former, in one sense, matter less, as no harm is done, but the latter may propagate errors into the public databases, and this in turn may cause experiments to fail. I will look in some detail at an example of this, and make some suggestions that could improve matters.

**Fig 3:**
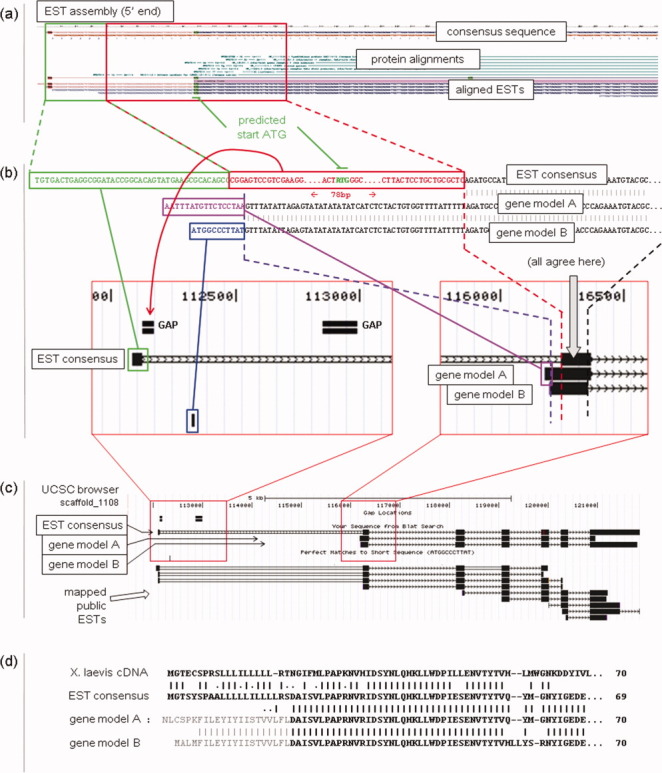
Mis-modeling of genes leads to incorrect sequences in public databases. There are gaps in the *Xenopus tropicalis* genome assembly in the ifngr2 gene locus, and this has created problems for the gene modeling process. The figure compares alignments for two gene models from different sources and two cDNA based sequences: *X. tropicalis* gene model A, Ensembl ENSXETT00000000072; gene model B, NCBI XM_002942799.1; EST assembly consensus, Gurdon Xt7.1-TNeu110f24.3; and *X. laevis* cDNA, NCBI NM_001099874.1. Panel (a) first 400 bp of the assembled EST contig. Visible striations in the EST alignments section indicate the quality of the assembly. The section of the contig sequence missing from the genome sequence is outlined in red. Panel (b) three-way alignment between the 5′-most parts of the EST consensus sequence and the two gene model sequences. Colored boxes and dashed lines link sections of sequence to their positions in the detailed view from the UCSC genome browser page. The red arrow indicates the most likely assembly gap to contain the ‘missing’ sequence. Panel (c) UCSC genome browser view for the whole ifngr2 locus on scaffold_1108, Xt-v4.1, showing BLAT alignments for the EST consensus and gene model sequences. Public EST alignments mapped by UCSC are also shown. Panel (d) a multiple sequence alignment of the resulting translated protein sequences (first ∼70 residues), including a full-length cDNA sequence from *Xenopus laevis*, showing where the gene model derived protein sequences diverge from the cDNA based sequences.

We have good expressed sequence data for the gene **ifngr2** in *Xenopus*, with a high quality EST assembly (Xt7.1-TNeu110f24.3) from *X. tropicalis* at least four sequences deep from independently sequenced clones, and a full-length cDNA sequence from *X. laevis*. There are also two gene model transcript sequences, one from Ensembl (ENSXETT00000000072) and one from NCBI RefSeq (XM_002942799.1). Unfortunately for the gene modeling programs which generated these sequences there are two gaps within the gene locus, and 118 bp of the EST contig sequence disappear into one or both of the gaps. There is no real reason to doubt the sequence of the EST data, and the overall situation is depicted in Figure 3. The section of the EST contig sequence which fails to map to the genome assembly contains the predicted start of translation, leaving only part of the 5′ UTR to be found upstream of the gap. This gives the gene modeling programs very little to get traction on. Both gene models wrongly (and differently) extend the second exon in the 5′ direction by 50 or more bp, with the NCBI gene model making a further 4 kb jump to a plausible (in the absence of the EST data) start codon. The NCBI gene model also appears to make a wrong choice at both ends of the second intron.

What are the consequences of this? Firstly, as the gene model sequences and the translated proteins derived from them are submitted to the public databases, they will appear authoritative, unless the investigator is familiar with the issues and chooses to look at the EST data. Second, experiments may be compromised by incorrect sequence data. For example, in the *Xenopus* community morpholinos are the tool of choice for gene knock-down, and they are usually designed to complement the sequence around the initiating methionine of a gene's mRNA. A morpholino design based on the gene models seen here would clearly not work. The ‘knock down’ would have no effect, the gene would be deemed nonessential to development, and a promising thread of investigation might be lost.

I can suggest some ways in which this problem may be approached, and I note that other abundantly gapped genome assemblies are likely to have similar problems. The longer term solution is to improve, or even ‘finish’ the genome, and that is a work in progress, which is discussed below. Another possibility would be to tinker with the gene modeling programs, essentially to make them more aware that they are dealing with an imperfect genome sequence and could make more use of EST data to avoid extending models in improbable ways. These programs already have the ability to use expressed sequence data as evidence for the *existence* of exons, but maybe do not place enough emphasis on the evidence for (or against) *connectedness* of candidate exons. In addition, this provides another rational for continuing efforts to collect full-length mRNA sequence for genes which do not have one.

We can also see the potential for knock-on effects of mismodeling of genes. Suppose one of the two **ifngr2** gene models described earlier was released some time before the other; long enough for the slightly wrong protein to become part of the public data set. This might then influence a second round of gene modeling by providing ‘evidence’ for part of a coding exon. In this case the use of protein databases to provide confirmatory evidence for exons could actually increase the likelihood of a second gene modeling program making a sub-optimal decision. There is a further recommendation suggested by this: that one should be wary of using same-species proteins as evidence of coding sequence, particularly if they are computationally generated, to support gene modeling.

**Fig 4:**
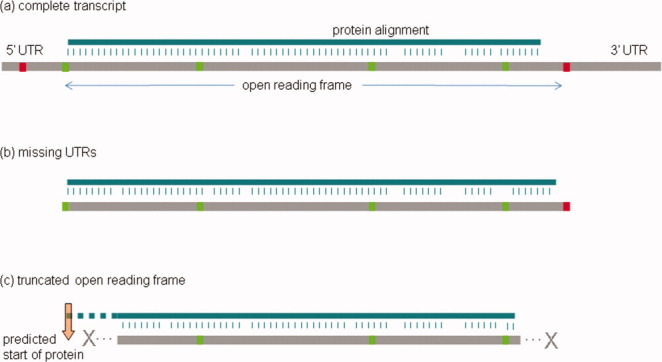
Systematic analysis for gene model transcript completeness. Transcript sequences are computationally analyzed (a) for the longest open reading frame (ORF), using in-frame ATG (green) and STOP codons (red). Missing UTRs are identified (b) where the ORF runs to the ends of the transcript. Truncated ORFs are identified (c) where protein matches are detected upstream of the most 5′ ATG and/or there is no 3′ STOP codon.

Problems with gene models can be detected analytically. Table 4 shows the results of an open reading frame (ORF) completeness test on v4.1 gene model transcripts downloaded from the Ensembl BioMart web site (Kinsella *et al*., 2011). The method of the analysis is illustrated in Figure 4. In addition, I used BLASTn to compare the gene models with a library of common repeats in *Xenopus tropicalis*, and classified model transcripts which were more than 80% covered by repeat sequence as probably not from true protein coding genes. The results for this assembly were somewhat surprising, but indicate that fewer than half the gene model transcripts (41%) have identifiably complete ORFs. The lack of UTR sequence is generally less troubling, although the 5′UTRs are often useful, as suggested above. This analysis may not be perfect, and indeed would not detect the problems with **ifngr2** reported above, but it does provide a useful measure by which to assess improvements to the genome assembly, and/or the workings of gene modeling programs.

**Table 4:** Analysis of Gene Model Transcript Sequences

v4.1	v7.1	Description
27,653	43,436	Total gene model transcripts
868	1056	80% high copy number repeat
3536	486	ORF truncated both ends
2307	1262	ORF truncated 5′
9594	2798	ORF truncated 3′
11,348	37,834	Total complete ORF transcripts
4020	4610	No UTRs
399	2920	No 5′ UTR
3230	3396	No 3′ UTR
3699	26,908	OK transcripts with UTRs

Available gene model transcripts from different sources were downloaded for the two versions of the *Xenopus tropicalis* genome assembly and analyzed for completeness of open reading frame (ORF), presence of UTR sequence, and repeat sequence composition. Top row shows the numbers of model sequences analyzed, followed by the numbers detected at decreasing levels of severity of problem. No sequence is reported in more than one error category.

## NEW GENOME AND TRANSCRIPTOME ASSEMBLIES

The comparatively incomplete nature of the genome assembly has been known for some time, and efforts have been underway to produce a new genome assembly. With the addition of a small amount of new sequence data, and a switch from the JAZZ assembler (Aparicio *et al*., 2002) used for v4.1 to the ARACHNE assembler (Batzoglou *et al*., 2002), a new version of the *Xenopus tropicalis* genome sequence was put together during 2010/11, and this has allowed the generation of a new set of gene models. The main features of this assembly, known as v7.1, are much improved long range contiguity, and a reduction in the number of gaps in the assembly by about 60–70%. The improvement in long range contiguity shows up in the chromosomal scale assembly of much of the genome. This was achieved by combining the sequence-based assembly process with information from the available genetic map along with syntenic alignments to the chicken genome. The new assembly and its associated gene model data are unpublished, and for the information presented and analyzed here I am grateful for personal communication from Richard Harland, Jerry Jenkins, Dan Rokhsar, Jeremy Schmutz, and Shengqiang Shu.

Although the number of gaps in the assembly has beendramatically reduced, there still exists scope for the disruption of the gene modeling process. Anecdotally, investigation of individual gene loci suggested an improvement in this respect, and to test this I applied the same analysis to the gene model transcripts from the v7.1 assembly as to the v4.1 model transcripts described earlier. I found a distinct improvement: the relative number of transcripts with apparently complete ORFs increased from 41 to 87% (see Table 4), and the absolute numbers increased from 11,348 to 37,834. It is conceivable that some of this increase is taken up by greater numbers of alternative transcripts for small numbers of genes, as opposed to additional numbers of modeled gene loci. I do not present any analysis at this level.

This genome assembly is now informally in the public domain, as are the current set of associated gene model transcript sequences. At the time of writing they may be accessed via Xenbase (Bowes *et al*., 2008): through the genome browser, through BLAST, or (in the case of the gene models) downloadable by ftp. The exact location and accessibility of these sequences may change over time.

In addition to these efforts with *Xenopus tropicalis*, work has started on both genome and transcriptome assembly for *Xenopus laevis* (J strain), taking advantage of the new HTS technologies. These developments will ultimately lead to a much improved understanding of the *X. laevis* gene repertoire, in particular the role and prevalence of surviving (posthybridization) homeolog pairs of genes, and the ability to define exon junction coordinates for morpholino design. Several labs are involved in this effort, and the following information derives from personal communication from Richard Harland, Dan Rokhsar, Jarrod Chapman, Christian Haudenschild, Edward Marcotte, John Wallingford, Asao Fujiyama, and Masanori Taira. A draft genome sequence has been assembled at the scaffold level, currently with coverage of over 2 Gb of assembled sequence, and work is continuing. To complement this short read data, a BAC sequencing project in Japan, using longer Sanger sequencing, is predicted to contribute upwards of 130,000 pairs of reads for a nominal 5× coverage. This should significantly enhance long-range assembly. In addition to these genomic data, RNA-Seq data generation is being coordinated and shared between labs in order to perform large-scale, *de novo* transcript assembly. This has currently generated over 60,000 sequence contigs, nearly half of which are over 1 kb in length. Not surprisingly, these various projects are generating significant interest in the community.

## THE FUTURE

The recent rapid development of HTS technologies has presented the *Xenopus* community with an interesting opportunity to take what were once community scale projects (the original *X. tropicalis* genome project, and the large-scale EST projects in both species), and migrate them to the level of investigator-led projects. This is how the *X. laevis* genome project described above is developing. In the same vein, there is now NIH funding for other genome-scale projects for *X. tropicalis*: to improve long range contiguity of the genome assembly through SNP detection and meiotic mapping (Harland/Rokhsar); and to improve genome coverage and continuity, specifically over gene loci, whilst also digging deep into the transcriptome to assemble more of the missing transcript sequences (Khokha/Cho/Gilchrist). In addition, a considerable amount of BAC end sequencing for *Xenopus tropicalis* has been done at Genoscope in France, which will support future assemblies. This has generated over 77,000 paired Sanger reads, at a nominal 6.7× coverage (Nicolas Pollet, personal communication).

Complementary to these projects, which concentrate on the sequence data in the databases, we are also now funded to produce the *Xenopus* Orfeome, a nonredundant collection of naked ORF, Gateway-cloned cDNA reagents (Stukenberg/Hill/Gilchrist). This project will benefit both from the existing physical resources and from the rapidly improving sequence resources described here. It should begin to generate tangible benefits to the *Xenopus* research community within a year or two. It is important, and timely, that this happens: as we have now seen, our repertoire of clone-based reagents is impressive, but still far from complete. It seems unlikely that any further large-scale EST projects will be undertaken, and this shuts down the traditional route for creating and identifying full-length clones.

In this context, one of the specific challenges facing this, and other, model organism communities is how best to harness the undoubted power of HTS, while retaining the information content of long read, long insert sequencing. One specific problem lies with alternative transcription, and the inability to link codon usage over longer lengths of transcript than the fragment sizes being sequenced (currently <1 kb on the Illumina platform; http://www.illumina.com). The other underlying difference is that the sequence fragments are not cloned before sequencing, and cannot easily be recovered and reused as reagents, or modified for further investigation. Of particular interest, in relation to this problem, are the single molecule sequencing technologies, like that recently brought to market by Pacific Biosciences of California (http://www.pacificbiosciences.com). Unlike the Illumina technology this does genuinely sequence single molecules, although the error rate of over 10% and the limited life of the component bio-molecules mean we appear to be still some distance away from long (multi-kb) runs of high accuracy sequence. Hybrid solutions might offer a way forward: for example using short Illumina RNA-seq reads to ‘decorate’ a longer PacBio cDNA sequence. Here the PacBio sequence would provide the long-range integrity, and the Illumina reads the accuracy.

Finally, our analyses have begun to show where tools, developed for the more complete genomes of human, mouse, fly and worm, might be tweaked to get better results in a world of large but somewhat incomplete genome sequences.

Things can only get better.
